# *d*‑Electron Heavy Fermion Behavior in a Near-Room-Temperature Polar Metallic Ferrimagnet: A Case of Mn_5_SiC

**DOI:** 10.1021/acs.chemmater.5c00868

**Published:** 2025-06-25

**Authors:** Zachary T. Messegee, Vasile Ovidiu Garlea, Igor I. Mazin, Seung Han Shin, Yan Xin, Hari Bhandari, Stuart Calder, Resham Babu Regmi, Nirmal J. Ghimire, Joon I. Jang, Xiaoyan Tan

**Affiliations:** † Department of Chemistry and Biochemistry, George Mason University, Fairfax, Virginia 22030, United States; ‡ Neutron Scattering Division, 6146 Oak Ridge National Laboratory, Oak Ridge, Tennessee 37831, United States; § Department of Physics and Astronomy, 6111 George Mason University, Fairfax, Virginia 22030, United States; ∥ Quantum Science and Engineering Center, George Mason University, Fairfax, Virginia 22030, United States; ⊥ Department of Physics, 35014 Sogang University, Seoul 04017, Republic of Korea; # National High Magnetic Field Laboratory, Florida State University, Tallahassee, Florida 32310, United States; ∇ Department of Physics and Astronomy, University of Notre Dame, Notre Dame, Indiana 46556, United States; ○ Stavropoulos Center for Complex Quantum Matter, University of Notre Dame, Notre Dame, Indiana 46556, United States

## Abstract

Polycrystalline Mn_5_SiC was synthesized by using a high-temperature solid-state method. Mn_5_SiC adopts a polar space group (*Cmc*2_1_) with six crystallographic Mn sites confirmed by X-ray and neutron diffraction, transmission electron microscopy, and second harmonic generation experiments. The complex crystal structure features edge-sharing trigonal prisms and icosahedra, as well as face/edge-sharing pentagonal prisms. Magnetic measurements indicate ferrimagnetic ordering with a transition temperature of 284 K. The ferrimagnetic structure (magnetic space group *Cm’c’*2_1_) was further identified by powder neutron diffraction, where collinear Mn spins align along the crystallographic *c*-axis. The refined magnetic moment for each crystallographic Mn site at 4 K is 1.8(2), −2.42(9), −1.72(8), 0.51(6), 0.50(4), and 1.7(2) μ_B_. Density functional theory calculations confirm both the metallic behavior and the ferrimagnetic structure observed experimentally and further provide insight into the observed Mn moment dependence across crystallographic sites. The resistivity and specific heat measurements and density functional theory calculations reveal a substantially large Kadowaki–Woods ratio of 5 × 10^–5^ μΩ·cm/(mJ/mol)^2^ and a many-body renormalization factor of 5.5, indicating the unusual heavy Fermion behavior in such an itinerant magnetic metal.

## Graphical abstract

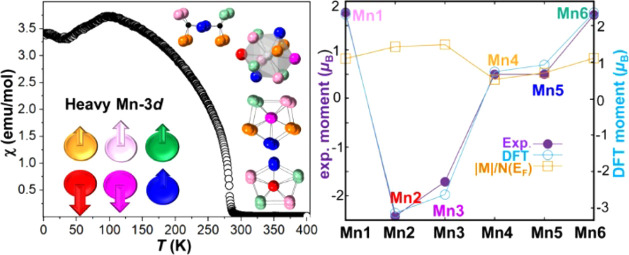

## Introduction

Crystal structures that lack inversion symmetry (noncentrosymmetric) and belong to specific point groups (1, 2, *m*, *mm*2, 4, 4*mm*, 3, 3*m*, 6, and 6*mm*) adopt polar space groups. Compounds that crystallize in such polar crystal structures and exhibit magnetic ordering can be defined as polar magnetic materials, which could show interesting and exotic electronic and physical properties that have potential applications in spintronics and quantum technology.^1–7^

Polar materials, as opposed to the centrosymmetric ones, have antisymmetric (Dzyaloshinskii–Moriya) exchange allowed on all bonds, rather than only on selected ones, and thus form fertile ground for nontrivial magnetic textures, such as helices and magnetic solitons (e.g., CrNb_3_S_6_),^8,9^ skyrmions (e.g., MnSi, GaV_4_S_8_, PtMnGa),^10–12^ and toroidal order (e.g., BaCoSiO_4_).^13^ Especially enticing is the possibility of combining ferromagnetism and ferroelectricity in multiferroic materials, which is only possible in a polar space group.^14,15^ Typical polar ferromagnets are metallic R_2_Ni_7_P_4_ (*R* = Ce, Pr, Nd; *Pmn*2_1_)^16^ and Weyl semimetals CeAlSi and PrAlX (X = Si, Ge) with low Curie temperatures (*T*_C_).^6,17,18^ The interesting magnetic behavior, transport properties, and topological features of polar materials inspired us to look for other polar magnetic intermetallics that have not been investigated in detail, especially those with high ferro- and ferrimagnetic (FM/FiM) ordering temperatures that could lead to practical applications at or above room temperature.

In this study, we present our investigation of FiM Mn_5_SiC, which shows a near-room-temperature magnetic ordering at 284 K and crystallizes in the polar space group *Cmc*2_1_.^19,20^ Interestingly, Mn_5_SiC nanowires have demonstrated magnetic hysteresis even at 400 K, nearly double the magnetic ordering temperature, and large magnetoresistance (∼80%) has been observed in the slightly boron-doped Mn_5_SiC nanowires.^21^ Mn_5_SiC is composed of earth-abundant, environmentally friendly, and economically viable elements, making it a promising candidate for future spintronics applications. Understanding the magnetic structure of this material is essential to its applications. However, despite powder neutron diffraction experiments in 1976,^20^ the magnetic structure had remained undetermined. Moreover, the electronic structure and other physical properties of this compound have not been reported. Here, we investigate the crystal structure and magnetic structure using powder neutron diffraction and reveal the physical properties by measuring the magnetic ordering, heat capacity, and resistivity. In addition, we use density functional theory calculations to study the electronic structure and provide insights into the observed physical properties in experiments.

## Experimental Section

### Starting Materials and Synthesis

Polycrystalline Mn_5_SiC samples were prepared by a conventional solid-state reaction. A stoichiometric mixture of Mn (Alfa Aesar, 99.95% metal basis), Si (Alfa Aesar, 99.999% mass fraction), and C black (Alfa Aesar, acetylene 100% compressed, 99.9+%) powders were weighed and ground thoroughly, which was pressed into a pellet (diameter = 6 mm). The pellet was transferred to a quartz tube that was sealed under a dynamic vacuum (<10^–3^ Torr). The obtained quartz ampule was heated to 1373 K at a rate of ∼114 K/h, dwelled at 1373 K for 1 week, and cooled to room temperature at a rate of 150 K/h. The dense pellet (92% density, diameter = 2.44 mm, height = 2.09 mm) was prepared by pressing Mn_5_SiC powders at 4 GPa and 673 K using a Walker-type high-pressure press. All sample preparation was conducted inside an argon-filled glovebox with a concentration of O_2_ and H_2_O less than 1 ppm, and no uncommon hazards were observed.

### Powder X-ray Diffraction

Powder X-ray diffraction (PXRD) patterns for polycrystalline samples were measured using a Rigaku Miniflex-600 benchtop X-ray powder diffractometer (Cu K_α_, λ = 1.5418 Å) by increasing the scattering angle 2Θ from 10° to 90° for 1 h.

### Second Harmonic Generation

A compressed pellet (∼6 mm) of Mn_5_SiC powders was used for second harmonic generation (SHG) measurements at room temperature in a backscattered geometry employing a homemade microscope setup. Excitation was achieved using an ultrafast Ti:sapphire laser with a pulse width of 100 fs and a repetition rate of 80 MHz, operating at an input wavelength (λ) of 800 nm. The SHG signal, with a wavelength (λ_SHG_) of λ/2 (400 nm), was collected by using a fiber-optic bundle coupled to a high-resolution spectrometer, which then directed the signal to a charge-coupled device camera. A long data collection time of 4 min was required to obtain the SHG signal well above the signal-to-noise level under an intense excitation level of 131.7 GW/cm^2^.

### Transmission Electron Microscopy

A probe-aberration-corrected sub-Å resolution JEOL JEM-ARM200cF microscope was used for all transmission electron microscopy (TEM) experiments with an accelerating voltage of 200 kV. Thin Mn_5_SiC pieces were ground from polycrystalline powders using a pestle and mortar and transferred to a carbon-coated 200-mesh Cu TEM grid. On a single thin piece, selected area electron diffraction (SAED) patterns were obtained along the [001] zone axis, and the corresponding atomic-resolution high-angle annular dark-field scanning transmission electron microscopy (HAADF-STEM) images were collected as well. Convergent-beam electron diffraction (CBED) patterns were collected by focusing the electron beam onto the thin piece with several tens of nanometer diameter areas in the TEM nanodiffraction mode.

### Powder Neutron Diffraction

Powder neutron diffraction (PND) experiments were performed using the HB-2A high-resolution powder diffractometer at the High Flux Isotope Reactor at Oak Ridge National Laboratory.^22,23^ Powder samples (∼2.8 g) were loaded into a cylindrical vanadium container (diameter = 8 mm) that was placed in a top-loading, closed-cycle refrigerator. The PND data were collected from 4 to 300 K with a wavelength of 1.54 and 2.41 Å. Rietveld refinements and data analysis of PND data were carried out using the suite of FullProf programs.^24^ Magnetic structure symmetry analysis was performed using the computational tools available at the Bilbao crystallographic server.^25^

### Chemical Analysis

The chemical compositional analysis of Mn_5_SiC was performed on a polished dense pellet with an Octane Elect Plus energy-dispersive X-ray (EDX) spectroscopy system, which is an accessory to a JEOL JSM-IT500HRLV scanning electron microscope (SEM). The SEM images and corresponding Mn, Si, and C elemental maps were collected with an accelerating voltage of 15 kV.

### Physical Properties

For magnetic measurements, polycrystalline Mn_5_SiC powders were first loaded into a polycarbonate capsule and sealed with Kapton tape. This capsule was inserted into a plastic straw, sealed with GE varnish, and then mounted in a Quantum Design DynaCool physical property measurement system (PPMS). Zero-field-cooled (ZFC) and field-cooled (FC) protocols were used to measure the magnetic susceptibility between 1.8 and 400 K with an applied magnetic field (*B*) of 0.1 T. The magnetic hysteresis was measured at 3, 100, 200, 300, and 330 K with *B* ranging to ±9 T. The resistivity and heat capacity were measured on the same polished piece of the pressed dense pellet using the PPMS. The resistivity data were collected from 2 to 380 K using a 4-probe method (an excitation current of 4 mA) with *B* = 0 T. The 25-μm Pt wires were used and affixed to the dense pellet with Epotek H20E silver epoxy. The heat capacity was measured from 5 to 330 K with *B* = 0 T.

### Electronic Structure Calculations

To gain more insights into the formation of magnetism in Mn_5_SiC, density functional theory (DFT) calculations were performed using the Vienna *ab initio* simulation package (VASP), implementing the projector-augmented wave (PAW) basis.^26^ The PAW–PBE (Perdew–Burke–Ernzerhof) pseudopotentials from the VASP library, Mn_pv, Si, and C_h were used. The experimental crystal structure was used throughout the calculations. Total energy calculations were performed using a 5 × 5 × 5 *k*-point mesh in the Brillouin zone, while for the density of states (DOS) calculations, a 15 × 15 × 15 *k*-point mesh was employed.

## Results and Discussion

### Synthesis and Chemical Analysis

Polycrystalline Mn_5_SiC powder samples were successfully prepared via the high-temperature solid-state method based on a previously reported heating profile.^19^ The temperature of the reaction can also be lowered from 1373 to 1323 K, and the purity is still retained. Room-temperature laboratory PXRD data of the prepared polycrystalline sample and dense pellet match the theoretical pattern of the orthorhombic crystal structure with the polar space group *Cmc*2_1_ (Figure S1). Chemical analysis of the dense pellet of Mn_5_SiC was performed by SEM-EDX (Figure S2), and the EDX elemental maps indicate a homogeneous distribution of the Mn, Si, and C elements. The molar ratio of heavier atoms Mn:Si is determined to be 5.1:1, which is close to the nominal ratio in Mn_5_SiC.

### SHG

To confirm the noncentrosymmetric nature of the title compound, SHG measurements were performed on the pellets made by compressing powder samples. With an input laser source wavelength (λ) of 800 nm, the SHG signal is observed at a wavelength (λ_SHG_) of λ/2 (400 nm) (Figure 1). In spite of the metallic properties of the title compound, a clear SHG signal was observed from the sample under intense optical excitation owing to a fast repetition rate (80 MHz) of our laser. The SHG signal from the surface of the sample was confirmed to be negligible, indicating that the SHG is driven by electric dipoles inside the medium. Therefore, the SHG signal was confirmed to be intrinsic to the sample, unambiguously demonstrating the noncentrosymmetric nature of Mn_5_SiC.

**Figure 1:**
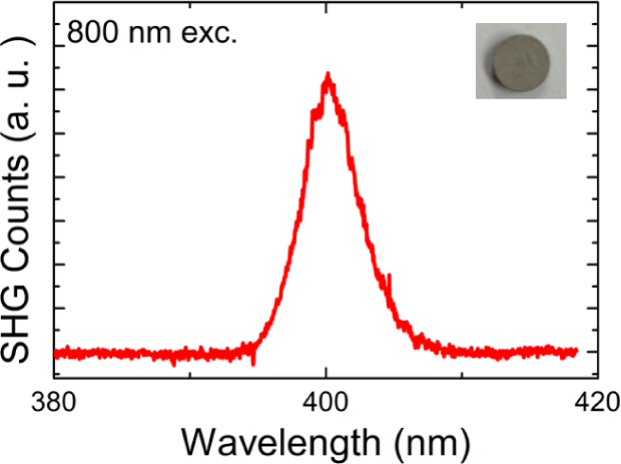
SHG spectrum observed from Mn_5_SiC. The inset shows a photograph of the pellet (diameter of ∼6 mm).

### PND and Crystal Structure

To refine the crystal structure, Rietveld refinements were performed using the PND data (300 K) with the previously reported orthorhombic crystal structure (space group *Cmc*2_1_) as the initial model. The final refinement indicates that this model fits the observed data well (Figure 2). A few extra tiny peaks at low angles (2Θ < 27°) are observed in the pattern, which we believe originate from impurities, but they cannot be identified based on all known possible binary/ternary compounds in the Mn–Si–C system. The selected corresponding structural parameters are listed in Table 1. In the crystal structure, there are six Mn sites (four Wyckoff positions 8*b* and two 4*a*), one Si site (Wyckoff position 8*b*), and two C sites (Wyckoff position 4*a*). The refined unit cell parameters [*a* = 10.2092(2) Å, *b* = 8.0455(1) Å, *c* = 7.6362(1) Å, and *V* = 627.23(1) Å^3^] are close to the reported values [*a* = 10.198 Å, *b* = 8.035 Å, *c* = 7.63 Å, and *V* = 625.21 Å^3^].^27^ The crystal structure was also confirmed at low temperature (4 K) based on the Rietveld refinement using the PND data, with the refined parameters shown in Table 1 for comparison. The unit cell parameters decrease as expected at a lower temperature, and the positions of all atoms are close.

**Figure 2:**
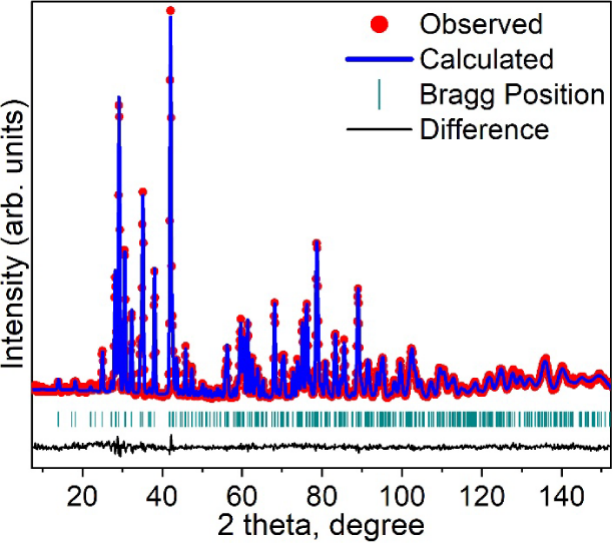
Rietveld refinement of Mn_5_SiC (space group *Cmc*2_1_) using PND data (300 K, λ = 1.54 Å) with observed data (red), calculated pattern (blue), Bragg peak positions (cyan), and the difference between the observed and calculated patterns (black).

**Table 1:** Selected Structural Parameters of Rietveld Refinements of Mn_5_SiC Using PND Data

sample	Mn_5_SiC	
temperature	300 K	4 K
mol. wt., g/mol	314.8	314.8
density (calculated), g/cm^3^	6.667	6.693
neutron wavelength	1.54 Å	1.54 Å
space group, #	*Cmc*2_1_, #36	*Cmc*2_1_, #36
*Z*	8	8
lattice parameters	*a* = 10.2092(2) Å, *b* = 8.0455(1) Å, *c* = 7.6362(1) Å, *V* = 627.23(1) Å^3^	*a* = 10.1897(2) Å, *b =* 8.0278(1) Å, *c =* 7.6217(1) Å, *V* *=* 623.465(1) Å^3^
Rietveld criteria of fit	*R*_p_ = 7.26%, *R*_wp_ = 7.86%, *R*_F_ = 2.08%	*R*_p_ = 7.87%, *R*_wp_ = 8.46%, *R*_F_ = 1.55%

site	Wyckoff symbol	*x, y, z*	*x, y, z*
Mn1	8*b*	0.1439(9), 0.3458(8), 0.3817(6)	0.1432(1), 0.3447(6), 0.3810(1)
Mn2	4*a*	0, 0.4538(6), 0.679(2)	0, 0.4540(4), 0.6783(7)
Mn3	4*a*	0, 0.1375(6), 0.189(4)	0, 0.1379(5), 0.1867(3)
Mn4	8*b*	0.1255(4), 0.004(2), 0.435(2)	0.1257(8), 0.0039(4), 0.4373(4)
Mn5	8*b*	0.1217(3), 0.1783(5), 0.695(3)	0.1215(1), 0.1780(4), 0.6924(1)
Mn6	8*b*	0.1499(9), 0.3319(9), 0	0.1506(5), 0.3324(1), 0
Si1	8*b*	0.2512(4), 0.0777(5), 0.188(3)	0.2512(1), 0.0780(1), 0.1902(2)
C1	4*a*	0, 0.194(1), 0.481(2)	0, 0.1952(3), 0.4795(1)
C2	4*a*	0, 0.191(1), 0.907(2)	0, 0.1895(1), 0.9059(3)

The crystal structure of Mn_5_SiC can be viewed as containing alternate “Slab 1” and “Slab 2” stacking along the *a*-axis, with Si atoms located between them (Figure 3a–d). The connection between Mn and C atoms is the same in “Slab 1” and “Slab 2”, but all atoms in “Slab 2” shift along the *c*-axis based on symmetry 2_1_ in comparison to the location in “Slab 1”. There are six Mn sites in the crystal structure. Viewing the “Slab 1” along the *a*-axis, the C atoms are located within a triangular prism to form a C@Mn_6_ trigonal prism (Figure 3e). Two C@Mn_6_ trigonal prisms are connected by edge-sharing of Mn5–Mn5, which can be defined as a C_2_@Mn_10_ “butterfly-like prism” with triangular bases (Figure 3e). The “butterfly-like prism” is connected into a three-dimensional (3D) network that is surrounded by pentagonal prisms consisting of Mn atoms in the *bc* plane (Figure 3e). Filling the Mn2 and Mn3 atoms in the pentagonal prism (forming Mn2@Mn_10_ and Mn3@Mn_10_) alternately along the *b* direction (Figure 3f) completes “Slab 1” (Figure 3g).

**Figure 3:**
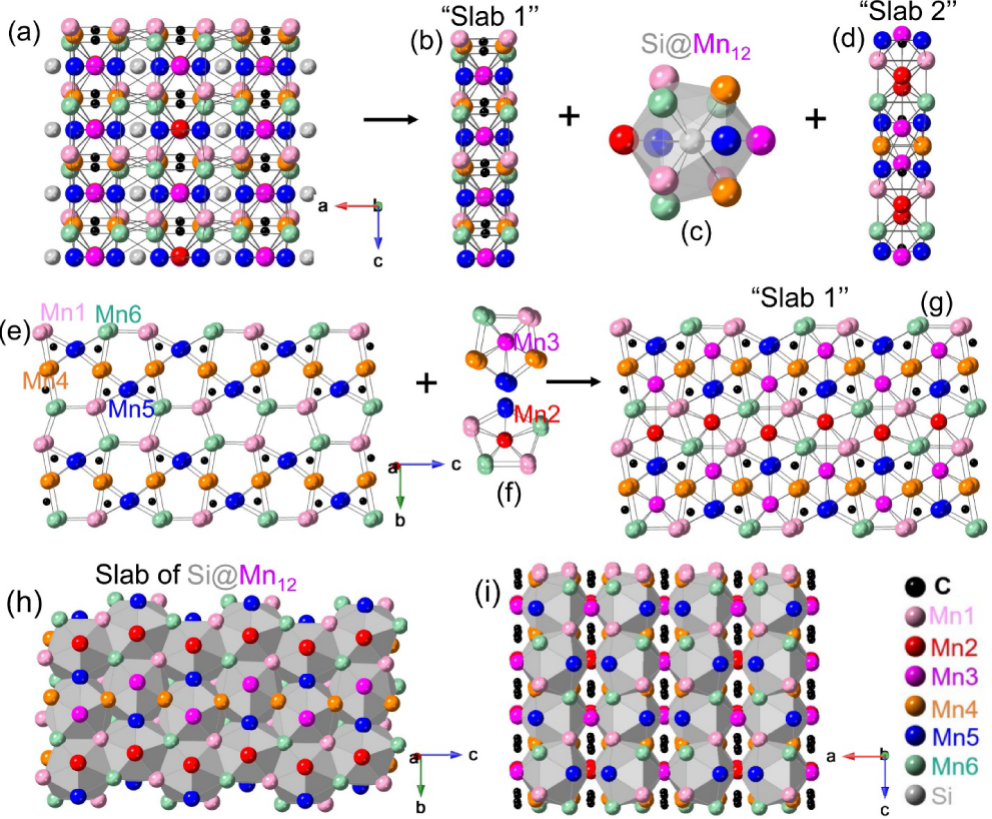
Perspective view of the crystal structure of Mn_5_SiC along the *b*-axis (a), “Slab 1” (b), a Si@Mn_12_ icosahedra (c), “Slab 2” (d), the 3D network of “butterfly-like prism” consisting of Mn1, Mn4, Mn5, Mn6, and C atoms (e), Mn2/Mn3-centering pentagonal prism (f), “Slab 1” along the *a-*axis (g), a slab of Si@Mn_12_ icosahedra along the *a*-axis (h), and the 3D network of Si@Mn_12_ connection (i). Figures of the crystal structure here and below were prepared using the CrystalMaker software.^28^

The Si atoms between “Slab 1” and “Slab 2” coordinate with 12 Mn atoms to form Si@Mn_12_ icosahedra (Figure 3c). In one Si@Mn_12_ icosahedron, each Si atom bonds with the pentagon of Mn atoms (Mn1–Mn6–Mn1–Mn5–Mn6) from the outer sheet of “Slab 1”, the other similar pentagon of Mn atoms (Mn5–Mn4–Mn6–Mn1–Mn4) from the outer sheet of “Slab 2”, and the Mn2 and Mn3 atoms, which are located inside the pentagonal prism (middle sheet) of both “Slab 1” and “Slab 2”. The two parallel pentagons are not the same and are twisted (not mirror images). The Si@Mn_12_ icosahedra with a coordination number (CN) of 12 is one of the typical features of tetragonally close-packed (TCP) geometries, in which the interstitial spaces are distorted tetrahedra.^29^ The Si@Mn_12_ icosahedra are connected via edge-sharing to form the slab of Si@Mn_12_ (Figure 3h), and the overall crystal structure contains layers of Si@Mn_12_ icosahedra with C atoms filling the holes of trigonal prisms (Figure 3i).

Figure 4 reveals more details of the prisms in the crystal structure. In the “butterfly-like prism”, each C@Mn_6_ trigonal prism contains different Mn–Mn bond distances, with the *d*(Mn–Mn) varying from 2.35 to 2.78 Å (Figure 4a). The bond distance between two identical Mn atoms varies from 2.563 to 3.06 Å. Therefore, these two triangular prisms contain scalene triangles (different lengths on three sides). As shown in Figure 4b, the C1 atom bonds with Mn1, Mn4, and Mn5 atoms with the corresponding *d*(C–Mn) equal to 2.05 Å, 2.02 Å, and 2.06 Å, respectively, while the C2 atom coordinates Mn4, Mn5, and Mn6 atoms with the corresponding *d*(C–Mn) all equal to 2.04 Å. The distances between C1/C2 and Mn2/Mn3 atoms in the neighboring pentagonal prism are C1/C2–Mn2 = 2.20/2.27 Å and C1/C2–Mn2 = 2.58/2.74 Å, respectively (Table 2). Considering those C1/C2–Mn2/Mn3 bonds, the two C atoms are connected with surrounding Mn atoms as a capped “butterfly-like prism”. In the Mn3@Mn_10_ pentagonal prism, *d*(Mn–Mn) between different Mn atoms varies from 2.35 to 2.918 Å (Figure 4c), while *d*(Mn–Mn) in the Mn2@Mn_10_ pentagonal prism varies from 2.54 to 3.05 Å (Figure 4d), which makes this pentagon base irregular. In the Si@Mn_12_ icosahedra (Figure 5a), the 12 *d*(Si–Mn) are different and are within the 2.39–2.91 Å range. The 20 triangles of the icosahedra with some of the Mn–Mn bond distances are shown in Figure 5b,c. The listed Mn–Mn, Si–Mn, and C–Mn distances in the crystal structure (Table 2) are comparable with those in known binary/ternary compounds in the Mn–Si–C system, such as Mn_3_Si,^30^ Mn_3_C,^31^ Mn_5_C_2_,^32^ Mn_7_C_3_,^32^ Mn_8_Si_2_C,^33^ Mn_16_SiC_4_,^34^ and Mn_17_Si_2_C_4_.^34^ Those compounds also share similarities in the connectivity of atoms in their crystal structures.

**Figure 4:**
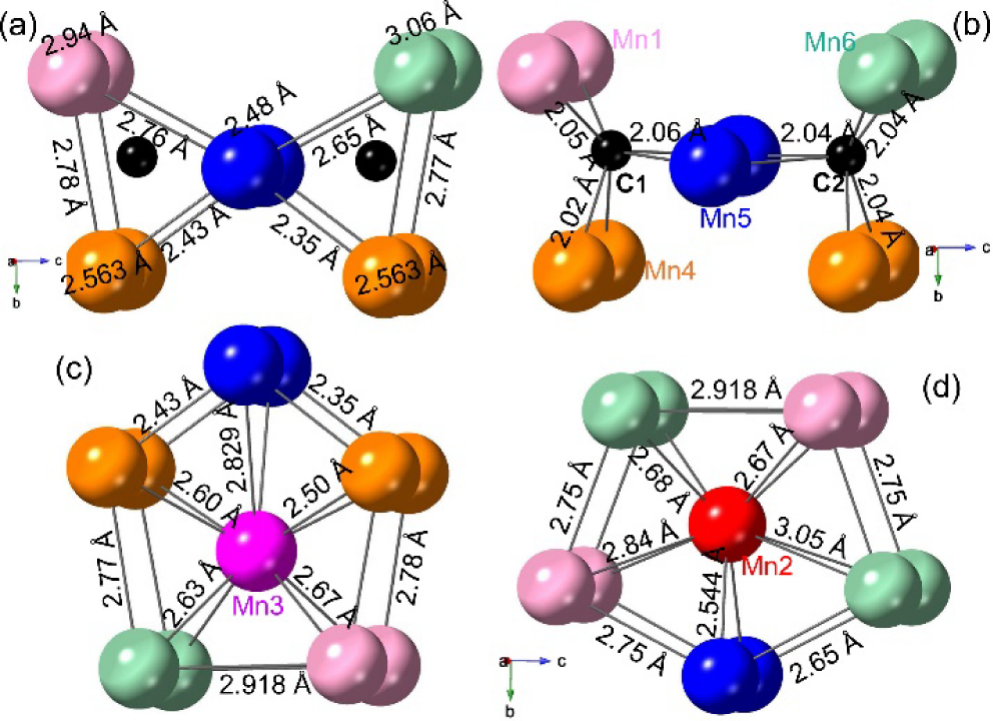
Perspective view of the C_2_@Mn_10_ “butterfly-like prism” labeled with Mn–Mn (a) and C–Mn bond distances (b), and Mn3@Mn_10_ (c) and Mn2@Mn_10_ (d) pentagonal prisms labeled with Mn–Mn bond distances.

**Figure 5:**
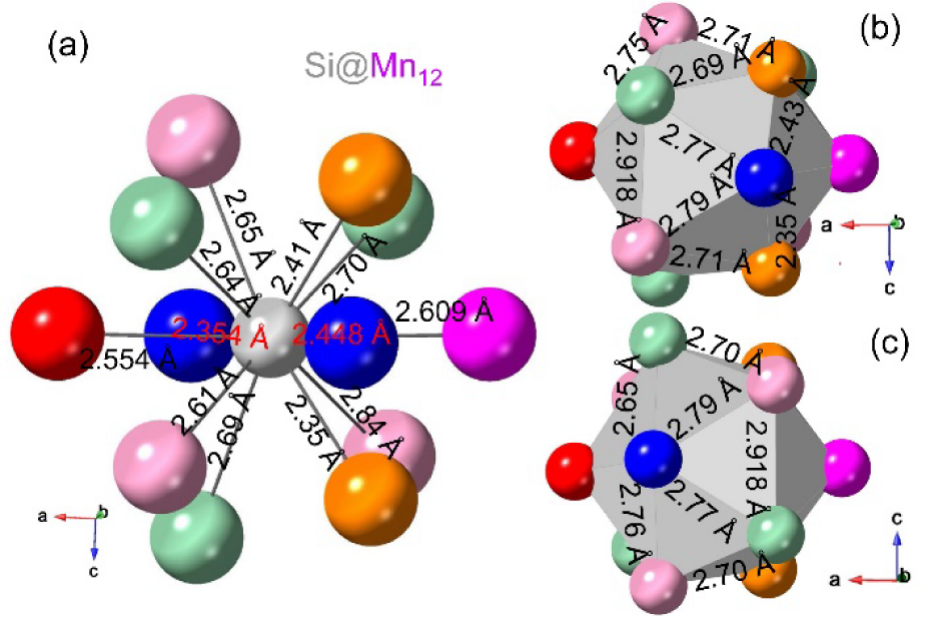
Perspective view of the Si@Mn_12_ icosahedra labeled with Si–Mn bond distances (a), and the Mn–Mn triangle surface of icosahedra viewed along the [010] (b) and [0 1̅ 0] direction (c) labeled with Mn–Mn bond distances.

**Table 2:** Selected Bond Distances of Mn_5_SiC Based on Rietveld Refinements Using PND at 300 K

Mn–Mn distances (Å)					
Mn4–Mn5 (×2)	2.35(2)	Mn1–Mn3 (×3)	2.67(2)	Mn5–Mn6 (×2)	2.77(1)
Mn4–Mn5 (×2)	2.43(2)	Mn1–Mn2 (×3)	2.67(1)	Mn1–Mn4 (×2)	2.78(1)
Mn5–Mn5	2.485(4)	Mn2–Mn6 (×3)	2.68(1)	Mn1–Mn5 (×2)	2.79(1)
Mn3–Mn4 (×3)	2.50(3)	Mn4–Mn6 (×2)	2.69(1)	Mn3–Mn5 (×3)	2.829(6)
Mn2–Mn5 (×3)	2.544(6)	Mn1–Mn6 (×2)	2.70(1)	Mn1–Mn2 (×3)	2.84(1)
Mn4–Mn4	2.563(6)	Mn1–Mn4 (×2)	2.71(1)	Mn1–Mn6 (×2)	2.918(5)
Mn3–Mn4 (×3)	2.60(3)	Mn1–Mn6 (×2)	2.75(1)	Mn1–Mn1	2.94(1)
Mn3–Mn6 (×3)	2.63(2)	Mn1–Mn5 (×2)	2.76(2)	Mn2–Mn6 (×3)	3.05(1)
Mn5–Mn6 (×2)	2.65(2)	Mn4–Mn6 (×2)	2.77(2)	Mn6–Mn6	3.06(1)
**Si–Mn distances (Å)**					
Si1–Mn5	2.354(6)	Si1–Mn2	2.554(4)	Si1–Mn1	2.65(2)
Si1–Mn4	2.35(2)	Si1–Mn3	2.609(4)	Si1–Mn6	2.69(2)
Si1–Mn4	2.41(2)	Si1–Mn1	2.61(1)	Si1–Mn6	2.70(1)
Si1–Mn5	2.448(6)	Si1–Mn6	2.64(2)	Si1–Mn1	2.84(1)
**C–Mn distances (Å)**					
C1–Mn4	2.02(1)	C2–Mn6	2.04(1)	C2–Mn3	2.20(3)
C1–Mn1	2.05(1)	C2–Mn4	2.04(1)	C1–Mn3	2.27(3)
C1–Mn5	2.06(2)	C2–Mn5	2.04(2)	C1–Mn2	2.58(1)
				C2–Mn2	2.74(2)

### Electron Diffraction

TEM experiments were performed on a thin piece of Mn_5_SiC to confirm the crystal structure with the polar space group *Cmc*2_1_. Along the [001] zone axis, the SAED pattern of Mn_5_SiC was collected and can be indexed according to the simulated pattern with the space group *Cmc*2_1_ (Figure 6a). The corresponding atomic-resolution HAADF-STEM image contains rows of bright spots with weak spots between those bright spots, as shown in Figure 6b. The intensity of the spot shown in the HAADF-STEM image is proportional to the atomic number (*Z* ^1.7^) of an atom, and the total number of atoms along the beam direction in the crystal structure being viewed. Therefore, the heavier the atom and the more atoms along that specific column at the same sample thickness, i.e., the larger the average atomic number per unit length, the brighter the spots. In the crystal structure of Mn_5_SiC, there are columns of Mn (*Z* = 25), Si (*Z* = 14), and C (*Z* = 6) atoms. Here, the crystal structure with space group *Cmc*2_1_ is viewed along the [001] zone axis (Figure 6c). Inside the projected unit cell, the Mn atomic columns labeled with Mn1–5 have Mn1 and Mn5 atoms alternating along the electron beam direction, and Mn3–3 atoms have Mn3 atoms along the beam direction, with both having a distance of 3.82 Å between Mn atoms along the electron beam direction. These are the densest columns with an atomic number per unit length of 6.54/Å, which corresponds to the brightest spots in Figure 6b. The weaker spots between those bright spots are the Mn columns of Mn2, Mn4, and Mn6 along the beam direction, which have an atomic distance of 7.63 Å along the beam direction with an average atomic number per unit length of 3.28/Å. The columns of C and Si atoms have an atomic number per unit length of 1.57/Å and 1.83/Å, respectively, which are too light to be observed in HAADF-STEM images, so they do not show any intensity in the image. It is clear that the atoms in the experimental HAADF-STEM image exactly match the atoms in the *Cmc*2_1_ unit cell.

**Figure 6:**
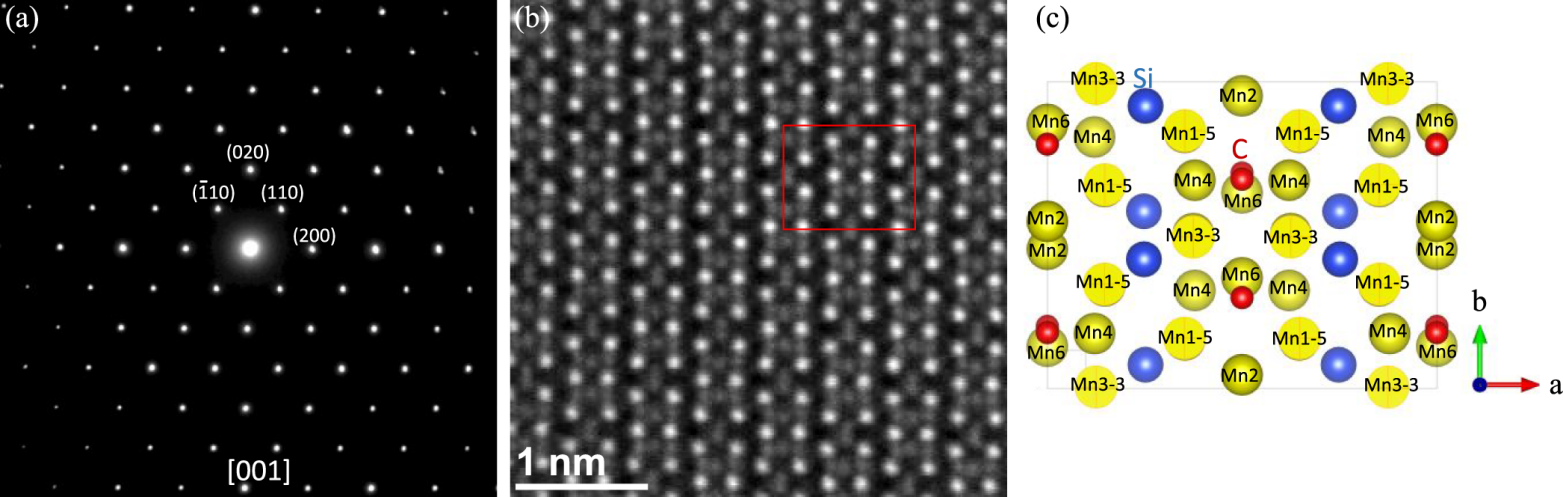
(a) Electron diffraction pattern along the [001] zone axis, (b) atomic-resolution HAADF-STEM image along [001], with the unit cell indicated by the red box, and (c) projected view of the unit cell of the crystal structure of Mn_5_SiC with the space group *Cmc*2_1_ along [001] (all the atoms along the beam direction are labeled, where, for example, Mn1–5 means that projected atomic column has Mn1 and Mn5 alternating along the electron beam direction, and Mn2 means that only Mn2 atoms are along the beam direction).

To further confirm the polar crystal structure, CBED patterns were collected since they are powerful in distinguishing the 32 point groups, which is related to dynamic scattering.^35–37^ The point group for *Cmc*2_1_ is *mm*2, and it is *mmm* for centrosymmetric *Cmcm*. For zone axis [uv0], the symmetry of the whole CBED pattern should have one mirror plane *m* for the point group *mm*2, while it should have a 2-rotation symmetry and two mirror planes *mm* for the space group *Cmcm*.^38^ The whole CBED pattern along the [120] direction is shown in Figure 7. Although it is very difficult to orient the crystal exactly down the zone axis and the diffraction disc intensity is very sensitive to the slightest mistilt, it is reasonable to say that we observe only one mirror plane as indicated by the yellow dashed line in Figure 7. The symmetry of the whole CBED pattern should be determined only by the whole pattern, i.e., by the zero-order Laue zone diffractions, as well as particularly by the symmetry of the high-order Laue zone diffraction pattern, such as in this case, the first-order Laue zone discs that form a ring around the zero-order diffraction discs in the center. After careful examination, the additional mirror plane and the 2-rotation symmetry are not observed in Figure 7. Thus, the overall TEM results confirmed the polar crystal structure with space group *Cmc*2_1_.

**Figure 7:**
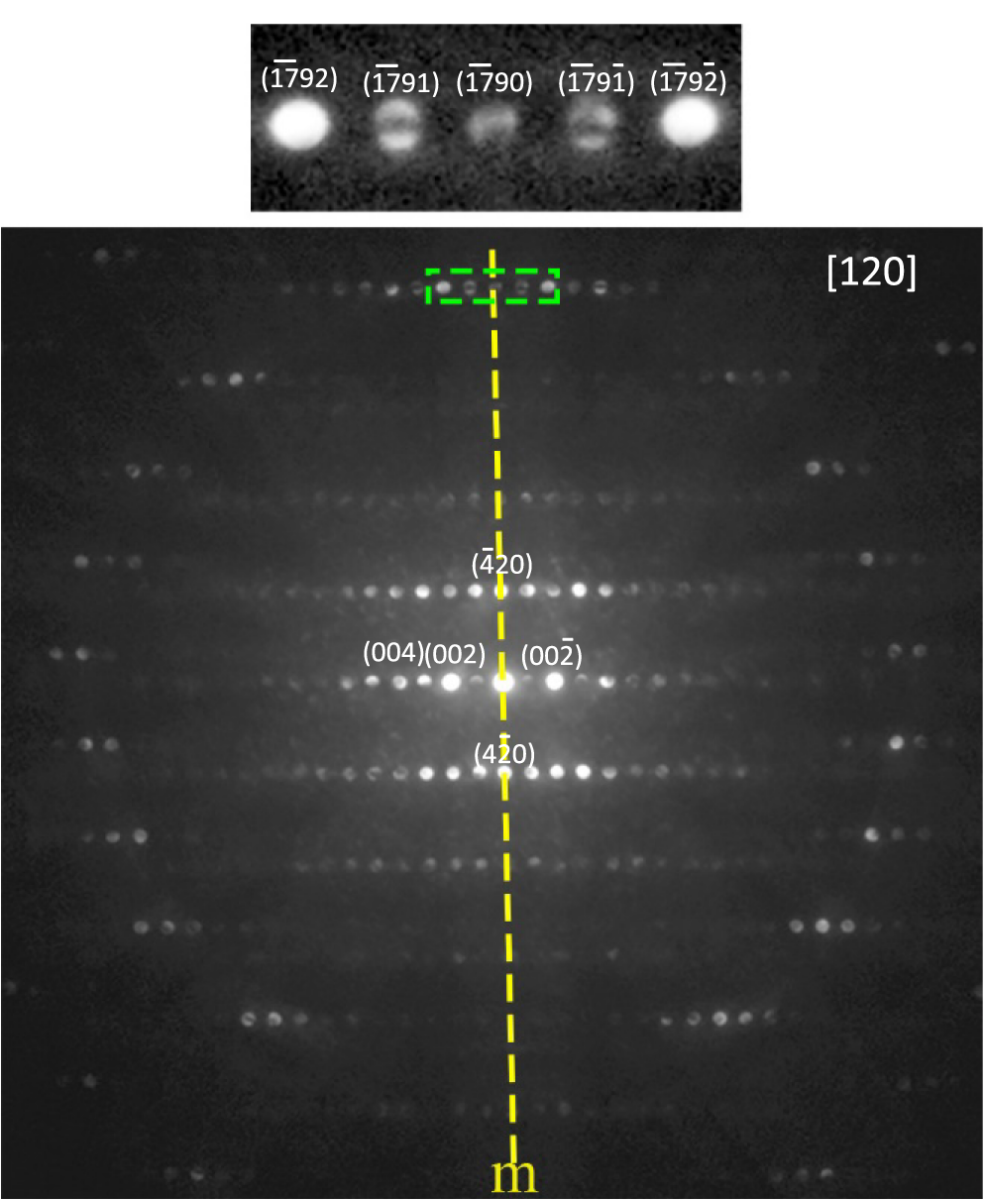
The whole CBED pattern along the [120] zone axis. The top figure is the zoomed-in part of the boxed top that shows the contrast inside the diffraction discs having mirror symmetry. The yellow line is a guide for the eyes.

### Physical Properties

The ZFC-FC magnetic measurements of Mn_5_SiC (Figure 8a) show a FM or FiM transition at 284 K. The FC/ZFC magnetic susceptibility decreases below 100/77 K and starts increasing below 37/35 K. The magnetic susceptibility data on the dense pellet show similar behavior (Figure S3), and our results are consistent with those of a previous report (Figure 8a).^20^ The high-temperature range (350–400 K) of ZFC data and inverse magnetic susceptibility (Figure 8b) were fit with the modified Curie–Weiss law (χ = χ_o_ + *C*/(*T* – θ_w_)), yielding the constant diamagnetic term (χ_o_) of 0.0016 emu/mol, Curie constant (*C*) of 0.51 μ_B_^2^, and a positive Weiss constant (θ_w_) of 284 K (Figure 8a). The value of θ_w_ is close to *T*_C_, and the large positive sign is consistent with the FM or FiM ordering. Note that such a simple Curie–Weiss theory is inapplicable to a system with several inequivalent magnetic atoms with distinctly different magnetic properties, and it is not formally applicable to the itinerant magnet.^39^ We will see further proof of that in the DFT calculations.

**Figure 8:**
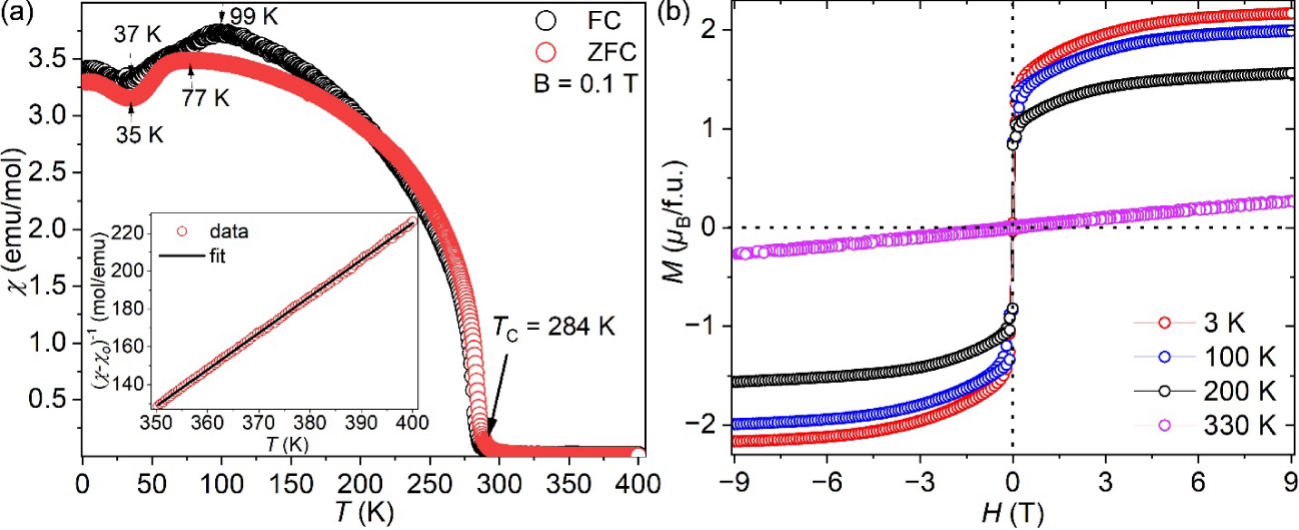
(a) Temperature-dependent ZFC-FC magnetic susceptibility of Mn_5_SiC at 0.1 T with inverse ZFC magnetic susceptibility fit with the 1/(χ – χ_o_) = (*T* – θ_w_)/*C* equation (inset) and (b) field-dependent (−9 to 9 T) magnetization of Mn_5_SiC at 3, 100, 200, and 330 K.

The isothermal magnetization as a function of the magnetic field was measured between 3 and 330 K, and it increases abruptly at low magnetic fields and reaches saturation at 9 T when the temperature is ≤200 K (Figure 8b). The saturated Mn moment at 3 K is 2.1 μ_B_/f.u. The hysteresis loops from −9 to 9 T show typical soft magnet behavior (Figure 8b).

Electrical resistivity as a function of temperature is depicted in Figure 9. The resistivity increases slightly as the sample is cooled from 380 K (2312 μΩ·cm) to about 202 K (2375 μΩ·cm), with a kink appearing near 284 K, which is likely associated with magnetic ordering (Figure 8). Below 202 K, the resistivity decreases with a decrease in temperature, indicating metallic behavior of the sample. The resistivity at 2 K is 638 μΩ·cm, which is relatively high for a metal. However, this value may have been influenced by the grain boundaries in the polycrystalline sample. The low-temperature behavior is perfectly quadratic. Fitting the low-temperature range (below ∼10 K) with the equation, ρ = ρ_o_ + *AT* ^2^, yields the coefficient in the quadratic term *A* = 0.4 μΩ·cm/K^2^.

**Figure 9:**
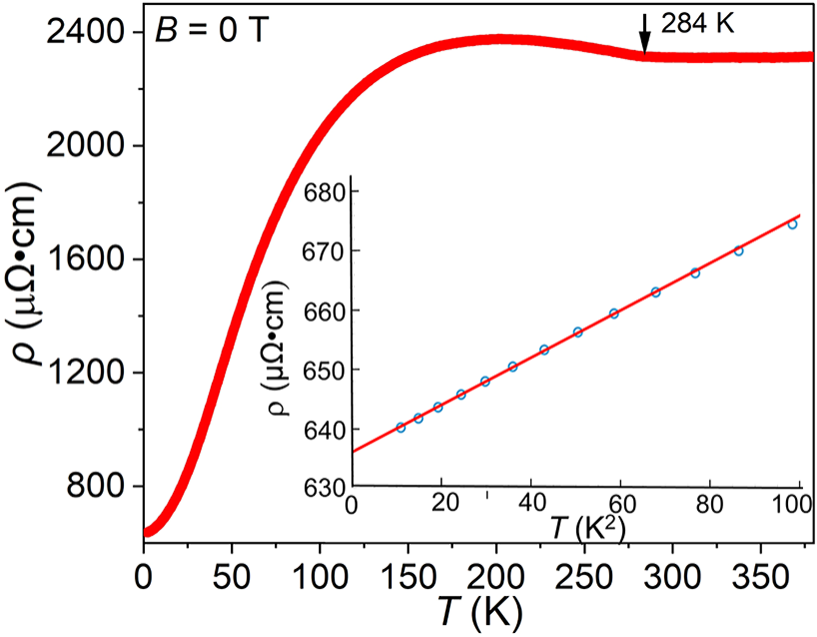
Temperature-dependent resistivity of Mn_5_SiC from 2 to 380 K with the low temperature fit with the ρ = ρ_o_ + *AT* ^2^ (inset). The arrow marks the Curie temperature.

Specific heat (*C*_P_) measurements show a λ-like anomaly at 278 K (Figure 10a), which is consistent with the long-range magnetic ordering observed in the magnetic susceptibility measurement (Figure 8). There is a small feature near 100 K, which may be related to the feature observed in the magnetic susceptibility in Figure 8a. The low-temperature data (5–21 K) can be fit well using the Debye formula, *C*_P_/*T* = γ + β*T* ^2^ to determine the value of γ and the lattice-specific coefficient β. The fit yielded Sommerfeld electronic specific heat coefficient γ = 83.13 mJ/(mol·K^2^) and acoustic phonon coefficient β = 2.12 × 10^–4^ J/(mol·K^4^). This γ corresponds to the electronic density of states at the Fermi level *N*(*E*_F_) of 35 states/(eV·f.u.). This is to be compared to the DFT-calculated value of 6.3 states/(eV·f.u.), implying a many-body renormalization factor of 5.5, which indicates the heavy Fermion (HF) behavior. Moreover, the Sommerfeld coefficient of 83.13 mJ/(mol·K^2^) is exceptionally large for typical 3*d* metals and comparable to that in so-called “*d*-electron heavy Fermion” compounds like Y_1 – *x*_Sc_*x*_Mn_2_ and Mn_3_P.^40–42^ The Kadowaki–Woods ratio,^43^ defined as *R = A*/γ^2^, is routinely used to quantify the “heaviness” of the Fermions, where *A* is the coefficient in the quadratic term in the fitting of low-temperature resistivity (0.4 μΩ·cm/K^2^ in our case). For heavy Fermions, *R* ≈ 10^–5^ μΩ·cm·mol^2^·K^2^/mJ^2^, and for regular transition metals, this value is ∼10 times smaller. In our case, *R* is 5.8 × 10^–5^ μΩ·cm·mol^2^·K^2^/mJ^2^, which is close to that of Y_1–*x*_Sc_*x*_Mn_2_, Mn_3_P, Mn_11_Ge_8_, and LiV_2_O_4_ (Table 3). Importantly, *d*-electrons typically show HF behavior near a quantum critical point when the long-range magnetic ordering is suppressed (thereby promoting spin fluctuations). Experimentally, such quantum critical materials should be a paramagnet or have a low transition temperature, as most examples shown in Table 3, except for Mn_11_Ge_8_, which shows FM ordering at 274 K and antiferromagnetic (AFM) ordering below the Néel temperature (*T*_N_) of 150 K.^44^ Here, Mn_5_SiC also quite uniquely approaches this regime despite strong ordered magnetism with near-room-temperature *T*_c_. This HF behavior is typically interpreted in terms of strong longitudinal spin fluctuations and/or magnetic frustration.^45^ Geometrical or magnetic frustration has been proposed to be the reason for the HF behavior in reported *d*-electron HF materials such as LiV_2_O_4_, Y_1–*x*_Sc_*x*_Mn_2_, β-Mn, YMn_2_Zn_20–*x*_In_*x*_, and Mn_3_P, which contain a tetrahedral lattice of Mn.^42,46,47^ In the example of LaMn_4_Al_8_, the arrangement of Mn atoms (1D nature) may be responsible for HF behavior.^48^ For Mn_11_Ge_8_, the existence of both localized and itinerant Mn moments of Mn and their interplay is proposed to be the reason for the spin fluctuations. The origin of HF in Mn_5_SiC may be related to the complex crystal structure and/or magnetic structure, which requires future systematic investigation based on single crystals.

**Table 3:** Summary of Mn-Containing *d*-Electron HF Materials Compared with LiV_2_O_4_

compound	space group	*T*_C_, *T*_N_	γ, mJ/(mol·K^2^)	*A,* μΩ·cm/K^2^	*R = A*/γ^2^, μΩ·cm·mol^2^·K^2^/mJ^2^	ref.
LiV_2_O_4_	*Fd* 3̅ *m*	Paramagnet	420, ∼350	2	∼10^–5^	^46,49^
Y_1–x_Sc_*x*_Mn_2_	*Fd* 3̅ *m*	Paramagnet	140, 150	0.25	1.1 × 10^–5^	^41,50,51^
β-Mn	*P*4_1_32 (NCS)	Paramagnet	70	-	-	^47,52,53^
LaMn_4_Al_8_	*I*4/*mmm*	*T*_N_ = 15 K	265	-	-	^54^
YMn_2_Zn_20–*x*_In_*x*_	*Fd* 3̅ *m*	Paramagnet	≥200	-	-	^55,56^
Mn_3_P	*I* 4̅ (NCS)	*T*_N_ = 30 K	104	0.52	4.8 × 10^–5^	^42^
Mn_11_Ge_8_	*Pnma*	*T*_C_ = 274 K, *T*_N_ = 150 K	139	0.42	2.2 × 10^–5^	^44^
Mn_5_SiC	*Cmc*2_1_ (NCS)	*T*_C_ = 284 K	83	0.4	5.8 × 10^–5^	our results

**Figure 10:**
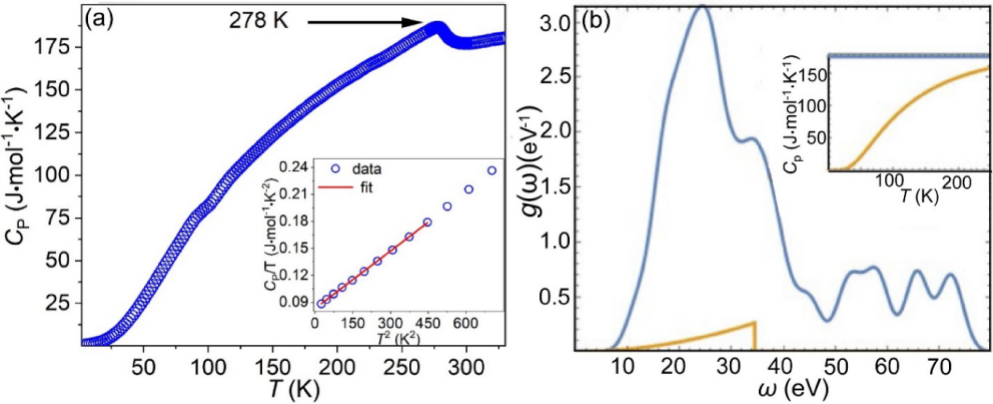
(a) Heat capacity of Mn_5_SiC measured from 5 to 330 K, with a low-temperature range (5–21 K) fit with the Debye relation *C*_P_/*T* = γ + β*T* ^2^ (inset). (b) Phonon density of states calculated using the DFT zone-center optical phonons (blue) and Debye phonons extracted from the low-temperature experimental data (yellow), and the inset shows the total phonon-only heat capacity compared to the Dulong–Petit limit.

Regarding the lattice-specific heat, the determined coefficient β corresponds to the acoustic Debye frequency θ_D_ of 400 K. At higher temperatures, the specific heat is mostly determined by the optical phonons. In order to estimate this contribution, we calculated DFT zone-center optical phonons and constructed the corresponding phonon density of states, as shown in Figure 10b. Using the acoustic and optical phonons together, the lattice part of *C*_p_ can be calculated, as shown in the inset. At *T* ∼ 280 K, it is still far from the Dulong–Petit limit of 175 J/(mol K) and noticeably lower than the experimental numbers. Comparing with the latter, we can assign the difference, ∼20 J/(mol K) below and 10 J/(mol K) above the transition to spin fluctuations.

### Magnetic Structure

To determine the magnetic structure, PND data were collected from 300 to 4 K. At 250 K, the intensity of most nuclear reflections at low angles increases compared to those of 300 K data (Figure 11a), such as (110), (200), (111), (020), and (021). The intensity of the (200) reflection shows the most dramatic change because of its pure magnetic nature. This peak keeps increasing below the magnetic ordering temperature, but there is no obvious change below 95 K (Figure 11b). The intensities of other reflections show only slight changes as the temperature decreases below 250 K (). The increase in the intensity of those reflections is contributed by the FM component from the magnetic structure. Considering the magnetic propagation *k*-vector (0, 0, 0) and the space group of the crystal structure, we tried the possible magnetic structure models with FM components along the *c-* and *b-*axes. The magnetic structure model with FM moments along the *a-*axis is excluded due to the existence of the (200) reflection. (Note that only spin components perpendicular to the scattering wave vector contribute to the magnetic neutron scattering.)

**Figure 11:**
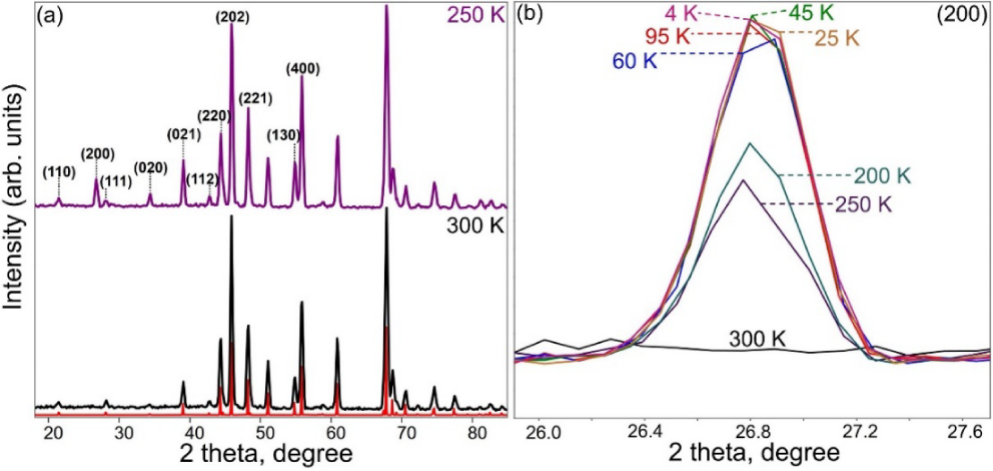
(a) PND patterns (λ = 2.41 Å) of Mn_5_SiC collected at 250 and 300 K (red pattern is the calculated one for comparison) and (b) a zoomed figure of the (200) reflection as the temperature changes.

The Rietveld refinements using PND data at 4 K indicate that only the magnetic structure model with a spin arrangement along the *c*-axis fits the data well (Figure 12). The refined magnetic space group (MSG) is *Cm’c’*2_1_ (#36.176), with Mn moment of 1.8(2), −2.42(9), −1.72(8), 0.51(6), 0.50(4), and 1.7(2)­μ_B_ for the Mn1–Mn6 site, respectively. The spin arrangement in the magnetic structure can be described as ↑↓↓↑↑↑, with the up arrow and down arrow indicating the opposite direction of the spin arrangement. In this collinear FiM magnetic structure, the magnetic moment on the Mn4 and Mn5 sites is much smaller than that on the other Mn sites. The MSG symmetry allows the alignment of Mn1, Mn4, Mn5, Mn6 magnetic moments (located on Wyckoff 8*b* site) in any crystallographic direction (i.e., (*M*a, *M*b, *M*c)), while the Mn2 and Mn3 moments (on Wyckoff 4*a*) are constrained to be in the *bc* plane (i.e., (0, *M*b, *M*c)). We also tried refining the data with a noncollinear magnetic structure, but the uncertainty of the Mn moments is as large as the refined value, which indicates that the collinear magnetic structure is the best fit within our experimental limits. The refined values for the collinear FiM model are provided in Table S1, and a full description of the magnetic structure, the magnetic cif file (mcif), is included in the Supporting Information.

**Figure 12:**
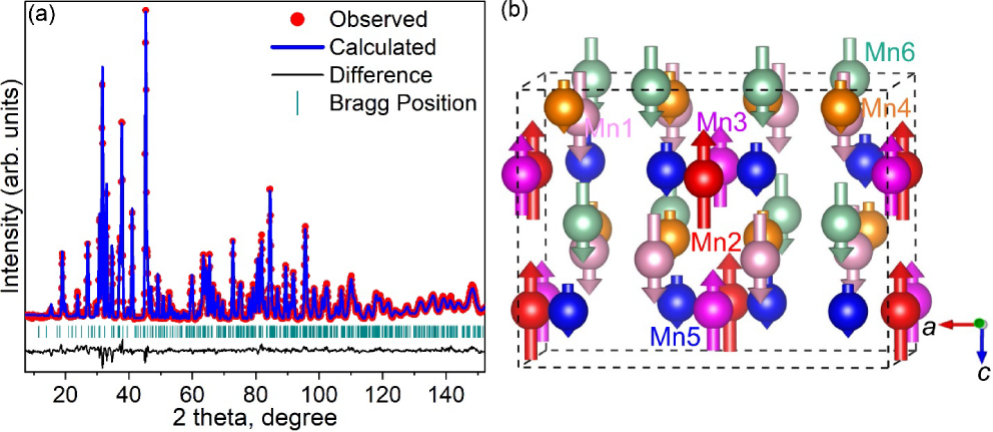
(a) Rietveld refinement of Mn_5_SiC (space group *Cmc*2_1_) using the PND collected at 4 K (λ = 2.41 Å) with observed data (red), calculated pattern (blue), Bragg peak positions (cyan), and the difference between the observed and calculated patterns (black). (b) Magnetic structure of Mn_5_SiC at 4 K (Si and C atoms are not shown for simplicity; the arrow represents the magnetic moments and spin orientation). The magnetic structure figure was prepared using the VESTA software.^57^

The FiM magnetic structure with different values of Mn magnetic moments on each crystallographic site has been observed in other binary and ternary Mn-containing compounds, such as Mn_2_Sb,^58^ Mn_1.9_Co_0.1_Sb,^59^ Mn_3_Al,^60,61^ Mn_3_In,^62^ Mn_5_Ge_2_,^63^ Y_6_Mn_23_,^64,65^ Er_6_Mn_23_,^66^ and LaNi_5–*x*_Mn_*x*_ (*x* = 1.5, 2).^67^ Interestingly, in this Mn_5_SiC system, most Mn–Mn bonds are shorter than 3 Å, and the only ones connecting strongly magnetic sites are Mn3–Mn6 (2.63 Å), Mn1–Mn3 (2.67 Å), Mn1–Mn2 (2.67 Å), Mn2–Mn6 (2.68 Å), and Mn1–Mn6 (2.70 Å), of which only the last one connects the same-sign moments, inconsistent with the large positive Curie–Weiss temperature given above. Again, this is a consequence of its itinerant character, which can generate relatively long-range magnetic interactions.

All other PND data collected at higher temperatures (25–200 K, λ = 2.41 Å) can be refined using the same nuclear and magnetic structures, as shown in Figure 12b. The refined moment for each Mn site (*M*_Mn_) and total magnetic moment (*M*_total_) are shown in Table 4 and Figure 12. The magnetic moment for all Mn sites increases as the temperature decreases from 250 to 60 K but shows slightly different behavior at lower temperatures (Figure 13a). Below 60 K, the magnetic moment difference between the maximum and the minimum is 15.8%–17.6% for Mn1, Mn5, and Mn6, and 3.6%–10% for Mn2, Mn3, and Mn4, forming two distinct groups. The *M*_total_ is 1.64(3) μ_B_/f.u. at 250 K and increases to its maximum of 2.49(3) μ_B_/f.u. at 60 K. The *M*_total_ then decreases to 2.00(9) μ_B_/f.u. at 45 K with a final upturn below 45 K and reaches 2.43(9) μ_B_/f.u. at 4 K (Figure 13a). The trend of the *M*_total_ moment changes as the temperature decreases, similar to the trend shown in the magnetic susceptibility (Figure 8a). The minimum in the magnetic susceptibility at *T* ∼ 35/37 K may be related to the local minimum in the magnetic moment, but the mechanism is unclear. Given the itinerant character of magnetism, it is likely related to spin fluctuations suppressing magnetism, with the temperature dependence of the fluctuation strength being different for different sites and the mean-field dependence of the magnetization.

**Table 4:** Refined Magnetic Moment for Each Mn Site and Total Magnetic Moment

^*T,* K^	^*M*^_^Mn1^_, ^μ^_^B^_	^*M*^_^Mn2^_, ^μ^_^B^_	^*M*^_^Mn3^_, ^μ^_^B^_	^*M*^_^Mn4^_, ^μ^_^B^_	^*M*^_^Mn5^_, ^μ^_^B^_	^*M*^_^Mn6^_, ^μ^_^B^_	^*M*^_^total^_, ^μ^_^B^_^/f.u.^
4	1.8(2)	–2.42(9)	–1.72(8)	0.51(6)	0.50(4)	1.7(2)	2.43(9)
25	1.6(2)	–2.46(9)	–1.71(7)	0.50(5)	0.46(5)	1.6(2)	2.09(9)
45	1.8(2)	–2.37(9)	–1.79(8)	0.45(6)	0.45(4)	1.4(2)	2.00(9)
60	1.9(1)	–2.45(1)	–1.73(7)	0.48(6)	0.54(5)	1.7(1)	2.49(3)
95	1.9(1)	–2.34(7)	–1.73(6)	0.43(4)	0.50(4)	1.6(1)	2.40(3)
200	1.4(2)	–2.08(8)	–1.19(7)	0.36(5)	0.36(4)	1.3(2)	1.83(9)
250	1.4(1)	–1.93(8)	–0.96(7)	0.32(5)	0.36(5)	1.0(1)	1.64(3)

**Figure 13:**
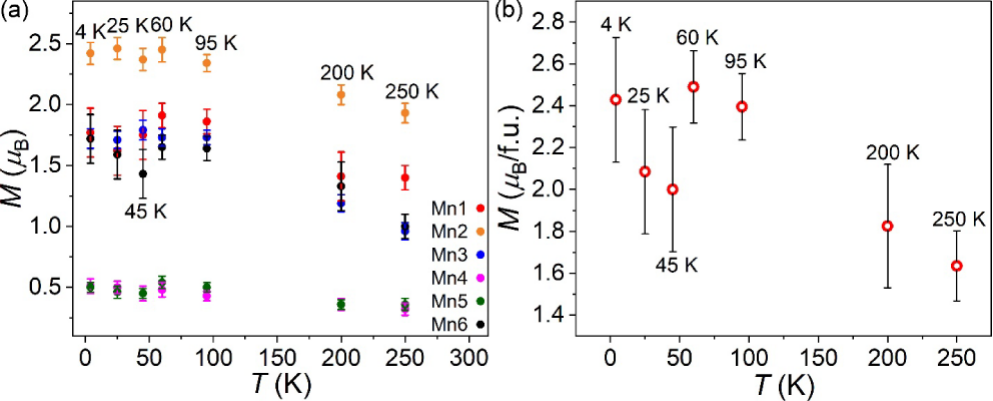
Refined magnetic moment for each Mn site (a) and the total magnetic moment (b) at different temperatures.

Based on the refined parameter of all PND data (4–300 K, λ = 2.41 Å), the unit cell parameters are plotted in Figure 14. There is no structural change as the temperature decreases, and the polar crystal structure remains. The lattice parameters decrease overall as the temperature decreases from 300 to 45 K, but parameters *a*, *b*, and *V* reach the lowest value at 45 K and show an upturn at lower temperatures, while parameter *c* behaves differently, increasing slightly at 45 K (Figure 14). The change in lattice parameter happens at 45 K, which coincides with the point when the total magnetic moment and magnetic susceptibility change, suggesting that there are spin–lattice couplings in the Mn_5_SiC.

**Figure 14:**
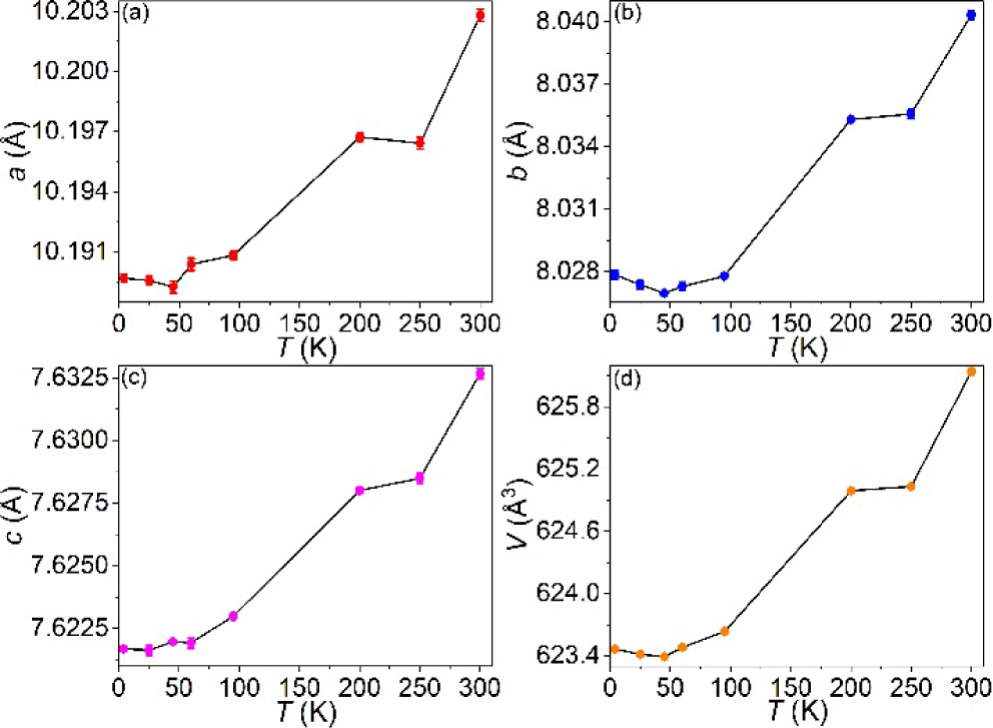
Unit cell parameters were obtained from the Rietveld refinement using PND data from 4 to 300 K. (The lines between the dots are just guides for the eyes.)

It is worth mentioning that three additional small peaks were observed below 45 K at 36.3°, 38.1°, and 40.3°, respectively (Figure S5). Those additional peaks may also be related to the dip observed in the magnetic susceptibility curve. However, it remains unclear whether they originate from the main phase or result from magnetic impurities. The magnetic propagation *k*-vector associated with those reflections cannot be uniquely determined, as no commensurate solution with the main phase was found. Moreover, no incommensurate *k*-vectors can be found for the [1,0,0] direction, consistent with the discussion in the 1976 report.^20^ Given the large number of nonequivalent magnetic ions (six Mn sites) in Mn_5_SiC and the limited number of observed peaks, any proposed model would be overparametrized and potentially misleading. We plan to pursue additional studies on the Mo-doped samples, similar to those conducted by Spinat and Herpin,^20^ to gain a better understanding of the origin of the low-temperature transition and to search for further evidence of a possible additional incommensurate order.

### DFT Calculations

To understand the determined magnetic structure based on PND data, we tried several different magnetic spin arrangements, and the one with the lowest energy is the same as the one observed in the experiment, namely, with the spin arrangement ↑↓↓↑↑↑ for Mn1–Mn6 atoms. The magnetic structure with a spin arrangement ↑↓↓↑↑↓ (or ↑↓↓↑↑↓) is 40 (or 50) meV/Mn higher in energy. Typically, for itinerant magnets, the experimental moments are systematically lower than the DFT ones by ∼30%.^39^ As shown in Figure 15a, DFT-calculated magnetic moments show the same trend as the experimental data; the difference between the theoretical and experimental values is about 30%. For itinerant magnets, the individual moments are also roughly proportional to the partial nonmagnetic density of states (DOS) at the Fermi level (*N*(*E*_F_)), in the spirit of the extended Stoner theory.^68,69^ Therefore, based on the calculated DOS for each Mn (Figure 15b), we plotted the ratio of the calculated magnetic moments to *N*(*E*_F_) for each Mn site, as shown in Figure 15a. The results indicate that those ratios are very close, which illustrates that the main reason for the moment disparity is the different participation of different Wyckoff positions at the Fermi level. Importantly, this explanation is valid only for fully itinerant magnetic metals, proving that the title system belongs to this class and is consistent with the analysis of the Curie–Weiss susceptibility above.

**Figure 15:**
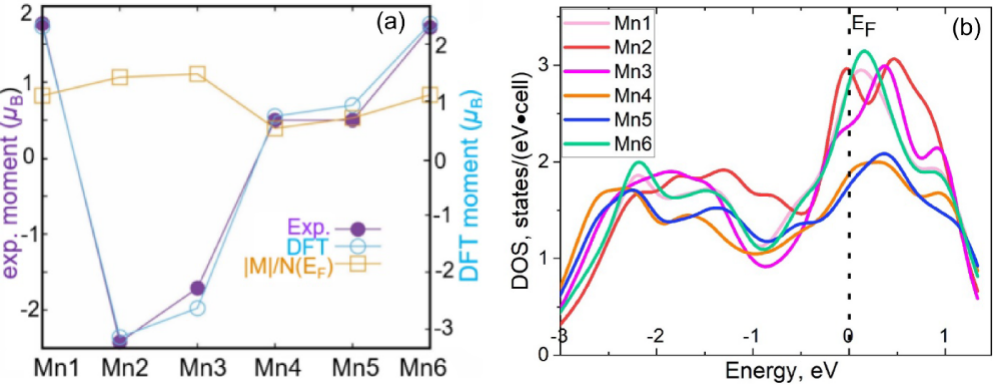
(a) DFT calculations of magnetic moments as compared with the experimental data, and the ratio of the calculated magnetic moment to *N*(*E*_F_) is plotted in arbitrary units. Note the different scales for the experimental and the calculated moments. The solid lines are guides for the eyes. (b) DOS plots for each Mn site in Mn_5_SiC.

We also performed a full optimization of internal atomic coordinates, keeping the unit cell dimensions consistent with the XRD data. Surprisingly, we found that the structure optimizes into a higher-symmetry centrosymmetric group, *Cmcm* (#63), with a considerable energy gain. The reason for this discrepancy is unknown at the moment. In any event, a loss of polarity would not affect the conclusions about the strong itinerant magnetism and heavy-Fermion behavior.

## Conclusion

Polycrystalline Mn_5_SiC was synthesized using the conventional solid-state method, and the polar crystal structure is confirmed. Based on the temperature-dependent PND data, the polar crystal structure remains the same in the temperature range of 300 to 4 K. The near-room-temperature FiM magnetic ordering is confirmed, and the determined FiM magnetic structure shows different magnetic moment values on each Mn site. The trend of total refined magnetic moment values matches tthat observed in magnetic susceptibility measurements. DFT calculations of the partial DOS and magnetic models reveal the reason for forming such a collinear FiM magnetic model with crystallographic site-dependent magnetic moment, which represents a good example for further understanding Mn-containing and transition metal intermetallics with FiM ordering. The results of Mn_5_SiC can provide guidance for investigating other polar metallic and magnetic materials. Transport and calorimetric measurements, combined with the DFT calculations, paint a unique picture of a very itinerant, on the one hand, but strong (i.e., with large, well-ordered moments) magnetism, on the other hand, which also shows an extremely strong many-body mass renormalization, approaching HF regimes, with an exceptionally large Kadowaki–Woods ratio. With this in mind, this compound holds unique promise as a platform for future research into strongly correlated spin-fluctuating itinerant metals.

## Supplementary Material





## Notes

PXRD patterns; EDX elemental maps; comparison of FC data; PND patterns (λ = 2.41 Å) collected between 4 and 300 K; zoomed-in PND patterns; and refined values for the collinear FiM magnetic model (PDF)
Full description of the magnetic structure (MCIF)
