# Supplementary material for: d‑Electron Heavy Fermion Behavior in a Near-Room-Temperature Polar Metallic Ferrimagnet: A Case of Mn5SiC
Source: Chem Mater. 2025 Jun 25;37(13):4832–43. doi: 10.1021/acs.chemmater.5c00868 (PMC12243080; doi:10.1021/acs.chemmater.5c00868)
Supplement: Supplementary file 1 [file cm5c00868_si_001.pdf]

d-Electron Heavy Fermion Behavior in a Near-Room-Temperature Polar Metallic Ferrimagnet: a Case of Mn_5SiC

Zachary T. Messegee,[†] Vasile Ovidiu Garlea,[‡] Igor I. Mazin,[§,‖] Seung Han Shin,[⊥] Yan Xin,[#] Hari Bhandari,[§%] Stuart Calder,[‡] Resham Babu Regmi,[%] Nirmal J. Ghimire,[%,§] Joon I. Jang,[⊥] and Xiaoyan Tan*,[†,‖]

[†]*Department of Chemistry and Biochemistry, George Mason University, Fairfax, Virginia 22030, United States*

[‡]*Neutron Scattering Division, Oak Ridge National Laboratory, Oak Ridge, Tennessee 37831, United States*

[§]*Department of Physics and Astronomy, George Mason University, Fairfax, Virginia 22030, United States*

[‖]*Quantum Science and Engineering Center, George Mason University, Fairfax, Virginia 22030, United States*

[⊥]*Department of Physics, Sogang University, Seoul 04017, Republic of Korea*

[#]*National High Magnetic Field Laboratory, Florida State University, Tallahassee, Florida 32310, United States*

[%]*Department of Physics and Astronomy, University of Notre Dame, Notre Dame, Indiana 46556, United States*

§ *Stavropoulos Center for Complex Quantum Matter, University of Notre Dame, Notre Dame, Indiana 46556, United States*

Corresponding Author* E-mail: xtan6@gmu.edu

Page

Figure S1. Experimental (Cu K_α, $\lambda = 1.5418$ Å) and calculated (space group $Cmc2_1$) PXRD patterns of Mn_5SiC S2

Figure S2. SEM-EDX elemental maps (a-c) of a Mn_5SiC pellet. S2

Figure S3. Comparison of temperature-dependent FC magnetic susceptibility of Mn_5SiC powder and dense pellet S2

Figure S4. PND patterns ($\lambda = 2.41$ Å) of Mn_5SiC collected between 4 and 300 K. S3

Figure S5. Zoomed-in PND patterns (33.5° < 2Θ < 45°, $\lambda = 2.41$ Å) of Mn_5SiC collected between 4 and 95 K S3

Table S1. Magnetic structure of Mn_5SiC described under MSG $Cm'c'2_1$, with basic information about its relation with its parent paramagnetic structure. S4

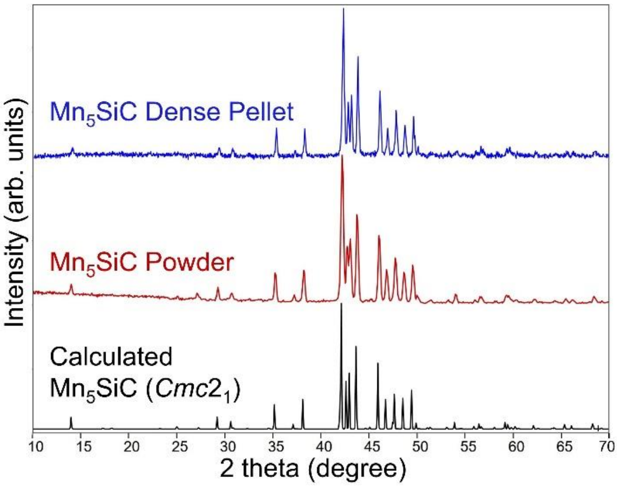

Figure S1. Experimental (Cu K_α, λ = 1.5418 Å) and calculated PXRD patterns (space group *Cmc*2_1) of Mn_5SiC.

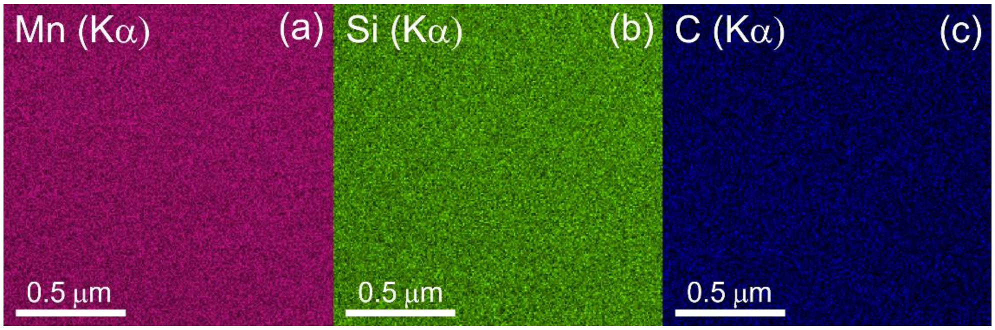

Figure S2. SEM-EDX elemental maps (a-c) of a Mn_5SiC pellet.

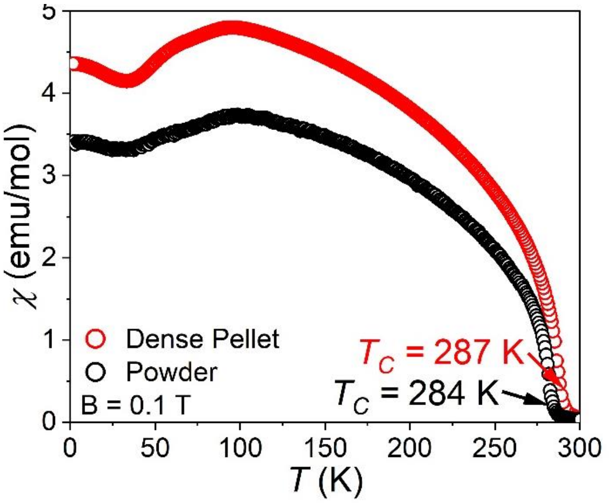

Figure S3. Comparison of temperature-dependent FC magnetic susceptibility of Mn_5SiC powder and dense pellet.

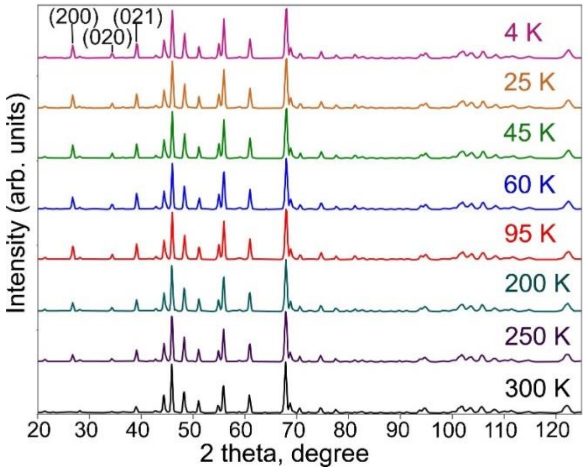

Figure S4. PND patterns ($\lambda = 2.41$ Å) of Mn_5SiC collected between 4 and 300 K.

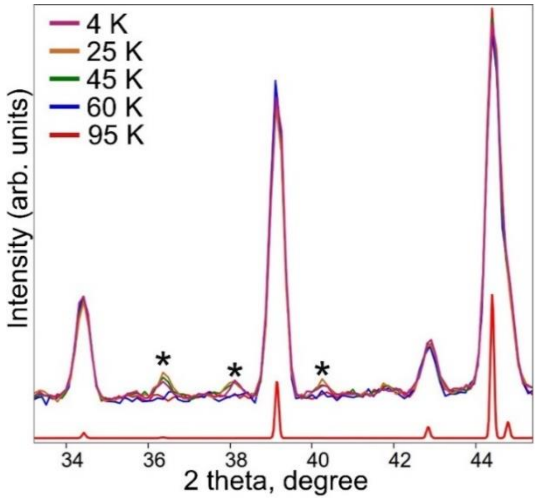

Figure S5. Zoomed-in PND patterns ($33.5° < 2\Theta < 45°$, $\lambda = 2.41$ Å) of Mn_5SiC collected between 4 and 95 K. Note that the origin of the small peaks in the 25 K and 4 K data remains unclear, as an inconmensurate k-vector that indexes these reflections cannot be uniquely defined. Considering the large number of non-equivalent magnetic ions (6 sites) in Mn_5SiC, and the very limited number of satellite peaks (3 peaks), any proposed model would be potentially misleading.

Table S1. Magnetic structure of Mn_5SiC described under MSG $Cm'c'2_1$, with basic information about its relation with its parent paramagnetic structure.

	Compound	Mn_5SiC
I.1	Parent space group	$Cmc2_1$
I.2	Propagation vector(s)	(0, 0, 0)
I.3	Transformation from parent basis to the one used for the magnetic structure	(***a***,***b***,***c***;0,0,0)
I.4	MSG symbol /number	$Cm'c'2_1$ (#36.176)
I.6	Transformation to standard setting of MSG	(a,b,c; 0,0,0)
I.8	Unit cell parameters (Å)	*a* =10.1897 α=90º<br>*b* = 8.0278 β =90º<br>*c* = 7.6217 γ =90º
I.9	MSG symmetry operations	x,y,z,+1<br>-x,y,z,-1<br>x,-y,z+1/2,-1<br>-x,-y,z+1/2,+1
I.10	MSG symmetry centering operations	x,y,z,+1<br>x+1/2,y+1/2,z,+1
I.11	Positions of magnetic atoms	Mn1 0.14321 0.34476 0.38100<br>Mn2 0.00000 0.45404 0.67837<br>Mn3 0.00000 0.13795 0.18673<br>Mn4 0.12578 0.00394 0.43734<br>Mn5 0.12150 0.17804 0.69239<br>Mn6 0.15065 0.33239 0.00000
I.12	Positions of non-magnetic atoms	Si1 0.25118 0.07808 0.19022<br>C1 0.00000 0.19523 0.47950<br>C2 0.00000 0.18949 0.90593
I.13	Refined magnetic moments components (μ_B) and, symmetry constraints	Mn1 0.00000 0.0(3) -1.8(2) (m_x,m_y,m_z)<br>Mn2 0.00000 0.0(2) 2.42(9) (0,m_y,m_z)<br>Mn3 0.00000 -0.0(3) 1.72(8) (0,m_y,m_z)<br>Mn4 0.00000 0.03(16) -0.51(6) (m_x,m_y,m_z)<br>Mn5 0.00000 -0.0(2) -0.50(4) (m_x,m_y,m_z)<br>Mn6 0.00000 0.0(3) -1.7(2) (m_x,m_y,m_z)
